# Emergence of carbapenem-resistant putative hypervirulent *Klebsiella pneumoniae* ST147 with a distinct hybrid plasmid in Türkiye

**DOI:** 10.1080/22221751.2026.2653280

**Published:** 2026-03-31

**Authors:** Cansel Vatansever, Anı Akpınar, Selin Belge, Nihan Kardan, Bade Tanyolaç, Selin Kolsuz, Jale Boral, Güz Ekinci, Burcu Işler, Önder Ergönül, Fusun Can

**Affiliations:** a Koç University İşbank Center for Infectious Diseases (KUISCID), Koç University, Istanbul, Türkiye; b Graduate School of Health Sciences, Koç University, Istanbul, Türkiye; c Department of Lymphoma & Myeloma, The University of Texas, MD Anderson Cancer Center, Houston, Texas, USA; d Department of Translational Medicine, Clinical Microbiology, Lund University, Malmö, Sweden; e Max Planck Institute of Infection Biology, Genetics of Host-Microbe Interactions, Berlin, Germany; f Infection Management Services, Princess Alexandra Hospital, Brisbane, Australia; g Department of Infectious Diseases and Clinical Microbiology, Koç University, School of Medicine, Istanbul, Türkiye; h Department of Medical Microbiology, Koç University, School of Medicine, Istanbul, Türkiye

**Keywords:** Carbapenem resistant, hypervirulent *K.pneumoniae*, ST147, hybrid plasmids, *bla*_OXA-48_, *bla*_NDM_

## Abstract

Carbapenem-resistant hypervirulent *Klebsiella pneumoniae* (CR-hvKp) represents a growing clinical threat due to convergence of resistance and virulence determinants. We conducted a multicenter genomic epidemiology study analysing CR-hvKp isolates collected from 19 tertiary-care hospitals across Türkiye between 2018 and 2023. CR-hvKp isolates were subjected to whole-genome sequencing to investigate resistance and virulence determinants, plasmid replicon profiles, and phylogenetic relationships. The proportion of CR-hvKp among CR-Kp increased from 20.7% in 2018 to 33.1% by 2023. We identified ST2096 as the predominant clone in 2018–2019, largely associated with *bla*_OXA-232_. However, after 2022, ST147 emerged as the dominant CR-hvKp lineage, characterized by co-carriage of *bla*_NDM-5_ and *bla*_OXA-48_, KL64 capsule, and exhibiting high antimicrobial resistance scores. Long-read sequencing revealed a 364-kb hybrid plasmid in an ST147 isolate, harbouring IncHI1B_pNDM and IncFIB_pNDM replicons. Notably, the ST147 hybrid plasmid (pPF302_hybrid) carried *rmtF1* and *arr-2*, resistance genes that have not been previously reported in hybrid plasmids linked to ST147. These findings indicate ongoing acquisition of resistance determinants in ST147 and highlight its increased adaptive potential under antimicrobial pressure. Our results demonstrate a clear clonal shift in Türkiye’s CR-hvKp population from ST2096 to ST147, alongside evolving resistance patterns and the emergence of hybrid plasmids. These insights emphasise the need for ongoing genomic surveillance and robust molecular characterisation in regions facing high CR-hvKp prevalence.

## Introduction

*Hypervirulent Klebsiella pneumoniae* (hvKp) has emerged as a major public health concern due to its ability to cause severe, often community-acquired infections in healthy individuals [1]. HvKp predominantly originated from South and Southeast Asia; however, cases attributed to hvKp are increasingly seen worldwide. Initially, hvKp strains were largely susceptible to antibiotics, but the increasing acquisition of multidrug resistance (MDR) plasmids in recent years has led to the emergence of carbapenem-resistant hvKp (CR-hvKp) clones [2–4]. In 2024, the World Health Organization (WHO) issued a global situation update highlighting the clinical importance of CR-hvKp [5].

The global prevalence of CR-hvKp has increased substantially in recent years, with rates ranging between 27.3% and 35.4% among clinical isolates reported from different regions worldwide [6–8]. In East and Southeast Asia, CR-hvKp prevalence has been reported as high as 63%, predominantly associated with ST11-KL64 and ST23-K1 lineages [9]. ST2096 has been reported as a major circulating clone in West Asia and the Middle East, including countries such as Türkiye, India, Jordan, and Saudi Arabia [10–12]. In Europe, hvKp was initially detected sporadically, and the most common ST types were ST395-K2, ST101, ST512, and ST147 [2]. Cross-border transmission has also been implicated in the dissemination of ST11-KL47 and ST147-KL67, suggesting the international spread of high-risk clones across the continent [2,13].

One of the key drivers of CR-hvKp’s global success is the emergence of hybrid plasmids that co-localize virulence and antimicrobial resistance determinants within a single backbone. These plasmids enable strains to exhibit both high-level drug resistance and enhanced pathogenicity, thereby increasing clinical severity and transmission potential [10,14]. Recent studies have identified such hybrid IncFIB – IncHI1B plasmids in ST147, ST2096, and ST383 hvKp isolates from India, carrying both virulence genes (*rmpA2, iutA, iucABCD*) and resistance determinants (*aadA2, armA, bla*_OXA-1_*, msrE, mphE, sul1, dfrA14*). These hybrid configurations likely contribute to the evolutionary success and expanding geographical range of CR-hvKp strains [10].

CR-Kp is highly prevalent in Türkiye, related to *bla*_OXA-48_ positivity; however, recently *bla*_NDM_ has also become frequently seen [15,16]. Considering the high burden of antimicrobial resistance, comprehensive genomic data on hvKp that have been circulating within the recent years remain limited. Despite increasing reports of CR-hvKp, the spatial and temporal dynamics of dominant clones and the genomic drivers underlying their geographic variation remain largely uninvestigated.

In this study, we aimed to characterize the genomic and epidemiological features of CR-hvKp isolates collected in Türkiye. By providing a detailed overview of their molecular profiles, including resistance, virulence determinants, and plasmid structures, we aim to improve understanding of CR-hvKp in this geographically strategic setting.

## Methods

### Study design, population, and isolate collection

This retrospective, multicenter molecular epidemiological study was conducted between December 2017 and December 2023 across 19 tertiary care hospitals located in five major regions of Türkiye (Istanbul, Aegean, Mediterranean, West Anatolia, and Western Black Sea). Isolates were included from two different studies, THREAT (NCT03597841) and KAPSAR. THREAT and KAPSAR were two prospective multicenter observational studies conducted during different time periods, but sharing identical study aims: to characterise clinical features and outcomes of patients with carbapenem-resistant *K. pneumoniae* bacteremia and pneumonia, and to identify predictors of mortality, with a focus on antimicrobial therapy. Representative isolates from each study period were included for genomic analysis. Participating centres were selected based on their microbiological expertise and prior experience in multicenter collaborations. Isolates from patients with bloodstream infections were obtained from blood cultures, whereas pneumonia-associated isolates were recovered from respiratory specimens (bronchoalveolar lavage, tracheal aspirates, or sputum), depending on routine clinical sampling practices at each participating centre. Exclusion criteria were incomplete data, polymicrobial bloodstream infections, or recurrent episodes in the same patient. A total of 599 non-duplicate CR-Kp isolates were collected and stored at the Koç University İşbank Center for Infectious Diseases (KUISCID). Ethical approval for the study was obtained from the Koç University Institutional Review Board (IRB Approval No: 2023.354.IRB2.071).

### Antimicrobial susceptibility testing and molecular detection of virulence determinants

All participating hospitals used different systems including MALDI-TOF MS, VITEK, and BD Phoenix systems for *Klebsiella pneumoniae* identification. Species-level confirmation was subsequently confirmed by whole-genome sequencing, and only isolates confirmed as *Klebsiella pneumoniae* were included in the final analysis. CR-Kp was defined as non-susceptible to at least one carbapenem (imipenem, meropenem, or ertapenem), according to EUCAST breakpoints [17]. Only carbapenem-non-susceptible isolates were included in this study. Minimum inhibitory concentrations (MICs) for meropenem, piperacillin-tazobactam, ceftolozane-tazobactam, and ceftazidime-avibactam, colistin were determined by broth microdilution using the Gram Negative EURGNCOL Plate (Thermo Fisher Scientific), and antimicrobial susceptibility profiles are provided in Table S2. Rifampicin susceptibility testing was performed for selected isolates using the disk diffusion method with rifampicin disks (Thermo Scientific™ Oxoid™, UK). The definition of putative hypervirulent *K. pneumoniae* (hvKp) was based on the presence of both siderophore and hypermucoviscosity-associated genes. Isolates carrying at least one aerobactin gene (*iucA* or *iutA*) and at least one mucoviscosity-associated gene (*rmpA* or *rmpA2*) were classified as hvKp [18,19].

### Whole genome sequencing and long read sequencing

Genomic analysis of 128 CR-hvKp isolates was conducted using whole-genome sequencing (WGS). Genomic DNA was extracted using the DNeasy UltraClean Microbial Kit (QIAGEN, Germany) from overnight cultures grown on Tryptic Soy Agar (Becton, Dickinson and Company, USA). DNA libraries were prepared using the Illumina DNA Library Prep Kit (Catalog #20091654). Library quality was assessed with the Agilent High Sensitivity DNA Kit, and quantification was performed using the QIAseq™ Library Quant Assay Kit. Sequencing was performed on the Illumina NovaSeq platform, generating 150-bp paired-end reads [20]. Nanopore long-read sequencing was performed for selected ST147 (n = 6) and ST2096 (n = 6) isolates, chosen among the distant representatives based on Mash pairwise distances. The DNA was extracted with Zymo Faecal Soil kits for Nanopore longread sequencing. The libraries were prepared with SQK-LSK114. Libraries were loaded into MinION flow cells R10.4.1 on a MinION MK1B. The sequencing run was conducted for 72 h with flow cell harbouring > 1400 pores at run start [21].

### Bioinformatics

Illumina raw read qualities were assessed with FastQC v.0.11.9 (https://www.bioinformatics.babraham.ac.uk/projects/fastqc/) and trimmed using TrimGalore v.0.6.10 (https://github.com/FelixKrueger/TrimGalore; – q 20 – stringency 5 – e 0.1 – length 30 – max_n 10). *De novo* assemblies were generated from trimmed reads using SPAdes v.4.0.0 ( – isolate, – plasmid) [22]. Assemblies were annotated with Prokka (https://github.com/tseemann/prokka, [23]) with default options for *K. pneumoniae*. Genome and plasmid assemblies were combined and processed with Kleborate v.3 for MSLT typing and major virulence factors [24]. Antimicrobial resistance genes were identified with AMRFinder v.4.0.3 [25] using Prokka-annotated genomes and the plus option. Plasmid incompatibility groups were identified using Abricate v.0.7 (https://github.com/tseemann/abricate, [26] – db plasmidfinder – minid 90 – mincov 80). For the phylogenetic tree, a pangenome was first built with panaroo v1.5.0 – clean-mode strict – refind_prop_match 0.5 – search_radius 1000) [27]. The clean core gene alignment by panaroo was then used to infer the phylogenetic relationships under the GTRGAMMA model with 20 bootstrap replicates using RAxML v.8.2.12 [28]. Visualisations were done with the interactive Tree of Life (iTOL v.7; https://itol.embl.de/) and Proksee [29]. All other analyses were done with custom scripts in Python.

For Nanopore plasmid sequencing data, raw read qualities were assessed with NanoPlot v.1.44.1 (https://github.com/wdecoster/NanoPlot, [30]) and cleaned with NanoFilt v.2.8.0 (https://github.com/wdecoster/nanofilt, [31], – quality 10, – length 1000). Assemblies were done with Flye v.2.9.5 (https://github.com/mikolmogorov/Flye; [32]) with – nano-raw option, followed by polishing with – polish-target. Assembled plasmid sequences were annotated with Prokka, as above [23]. Predicted transmissibility of the plasmids was assessed with the mob-typer tool from the MOB-Suite v.3.1.8 (https://github.com/phac-nml/mob-suite; [33]). Mash distances were calculated using Mash v2.3 (https://github.com/marbl/Mash).

## Results

### Sequence type distribution and phylogenetic relatedness of CR-hvKp isolates in Türkiye (2017–2023)

Between 2018 and 2023, a total of 599 CR-Kp isolates were identified, of which 78.1% were associated with bloodstream infections (BSIs) (Supplementary Table S2). Among these, 180 isolates (30.1%) were classified as CR-hvKp (Supplementary Table S1). The proportion of CR-hvKp among CR-Kp isolates was 20.7% in 2018, increasing to 33.1% by 2023.

Twelve distinct sequence types (STs) were identified among CR-hvKp isolates. The three most prevalent STs were ST2096 (n = 70, 54.7%), ST147 (n = 29, 22.7%), and ST383 (n = 11, 8.5%). Eight additional STs were represented by only one or two isolates each (Supplementary Table S1). ST2096 (92.9%, 65/70) was the most frequently identified genotype in BSIs, whereas ST147 (41.4%, 12/29) was predominantly associated with pneumonia cases. Genome distances did not suggest widespread clonality or assumed outbreaks (Figure S1).

The distribution of sequence types among CR-hvKp isolates varied over time. ST2096 was the dominant type in 2018, accounting for 91.3% (21/23) of CR-hvKp isolates in that year. However, its prevalence declined to 25% (8/32) by 2023. ST147 was not detected in 2018 and 2019 but emerged in 2022. Between 2022 and 2023, ST147 accounted for 40.8% (29/71) of CR-hvKp isolates (Table S1).

**Figure 1.**
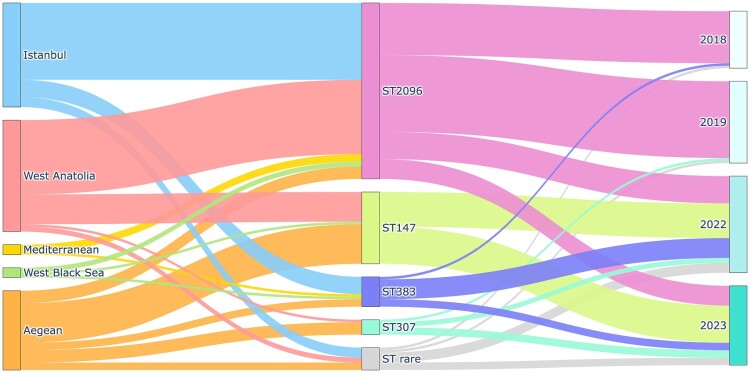
Regional distribution of sequence types in CR-hvKp*.* Sankey diagram illustrating the distribution of CR-hvKp sequence types (STs) across five regions of Türkiye (Istanbul, West Anatolia, Aegean, Mediterranean, and West Black Sea) and their temporal occurrence between 2018 and 2023.

A distinct geographical distribution pattern was observed for CR-hvKp sequence types across Türkiye (Figure 1 and S2). ST2096 was predominantly found in the İstanbul (43.6%) and West Anatolia (42.2%) regions (Figure 1). In contrast, ST147 was mainly identified in the Aegean region (55.2%) and West Anatolia (37.9%) (Figure 1 and S2).

### Virulence profile of CR-hvKp isolates

We constructed a maximum likelihood phylogenetic tree to assess the genetic relatedness and population structure of CR-hvKp isolates. The resulting tree revealed distinct and well-supported clades corresponding to individual sequence types (STs). The distribution of capsular (K) and lipopolysaccharide (O) loci, along with major virulence determinants (yersiniabactin, colibactin, and peg-344), was also mapped onto the phylogeny.

**Figure 2.**
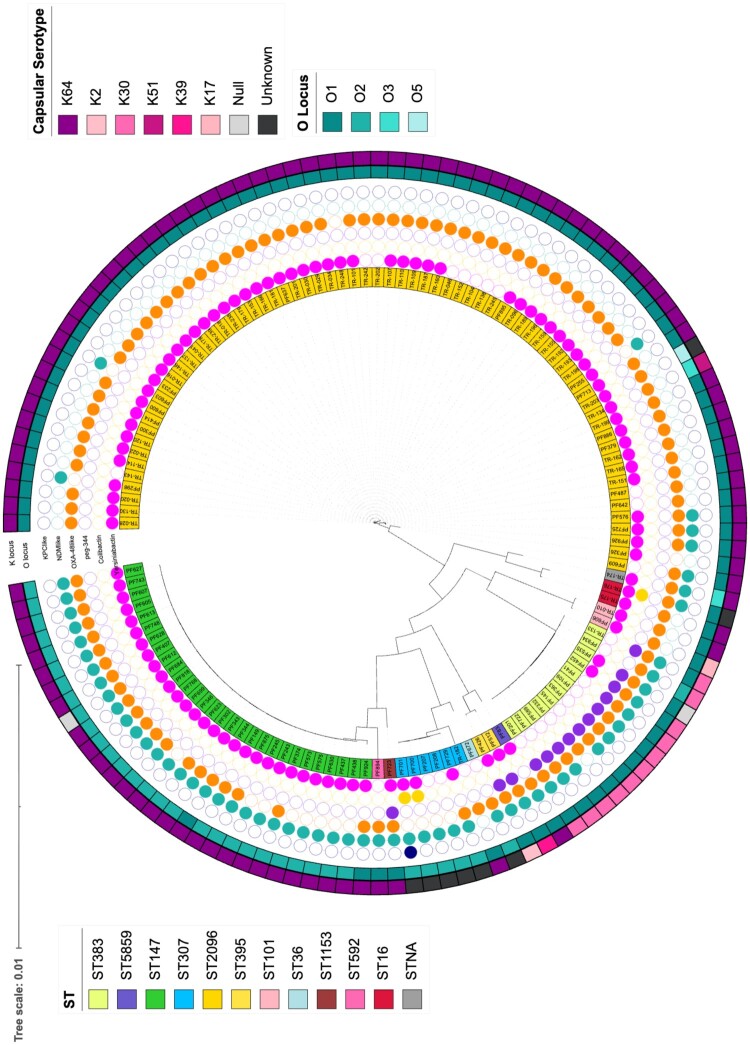
Phylogenetic analysis and virulence-associated features of CR-hvKp isolates. Maximum likelihood phylogenetic tree of CR-hvKp isolates based on core gene alignments. The innermost coloured ring denotes the multilocus sequence type (ST), with each colour corresponding to a different ST as indicated in the legend. The three tracks display the presence (filled circles) or absence (empty circles) of major virulence determinants: yersiniabactin (pink), colibactin (yellow), and peg-344 (purple). The outer second ring represents the capsular (K) locus type, and the third ring indicates the O-antigen serotype.

Genomic analysis revealed that 79.7% (102/128) of the CR-hvKp isolates harboured the chromosomal yersiniabactin (*ybt*) locus (Figure 2). All ST147 isolates carried the ybtS gene, which was associated with the yersiniabactin pathogenicity island of the ybt-9 lineage (Table S1). Among ST2096 isolates, 85.7% (60/70) were positive for the ybtS gene, all of which were linked to the ybt-14 lineage (Figure 2 and Supplementary Table S1). The colibactin locus (*clb*) was detected in only three isolates, and the salmochelin locus (*iro*) was not identified in any of the CR-hvKp isolates (Figure 2 and Table S1).

Capsular typing revealed six distinct K (capsular) loci, with K64 (79.2%) and K30 (8.4%) being the most prevalent. All of the ST147 and 97% (68/71) of the ST2096 isolates were associated with the K64 capsular type, whereas K30 was primarily identified in ST383 isolates (11/12, 91.7%). Regarding O-antigen types, the O1 antigen was detected in 100 isolates (78.1%), followed by O2, found in 35 isolates (27.3%). Notably, 97.1% of ST2096 isolates (68/70) carried the O1 antigen, while 93.1% of ST147 isolates (27/29) harboured the O2 antigen. The virulence-associated plasmid gene *peg-344* was present in 17 isolates (13.3%), the majority of which (11/17) belonged to ST383 (Figure 2). Tellurium resistance was detected in 93.7% (120/128) of CR-hvKp isolates, occurring in all ST147 isolates (100%, 29/29), in 97.1% of ST2096 isolates (68/70), and in 66.7% of ST383 isolates (8/12) (Table S1).

**Figure 3.**
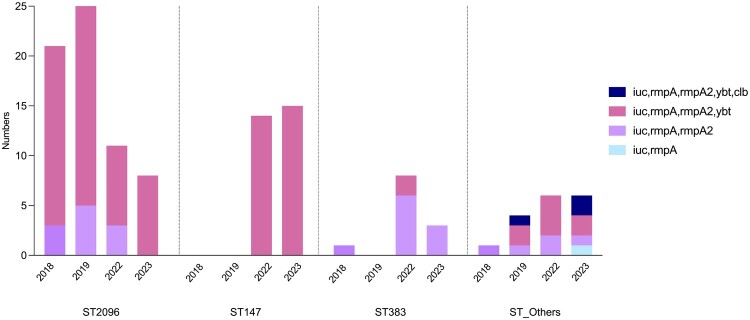
Distribution of virulence genes among CR-hvKp isolates by sequence type (2018–2023). Stacked bar chart showing the number of CR-hvKp isolates carrying virulence-associated genes per year, stratified by sequence type.

The distribution of hypervirulence-associated genes was analysed over time across major sequence types (STs). Among ST2096 isolates, most strains between 2018 and 2022 carried yersiniabactin (*ybt*) in addition to *rmpA/A2* and *iucA/iutA*, while a minority lacked it. In 2023, all ST2096 isolates were *ybt-*positive. ST147 isolates, first identified in 2022, consistently carried *ybt* in both 2022 and 2023. Co-occurrence of *ybt* and *colibactin* (*clb*) was observed only in less frequent sequence types, specifically ST307 and ST16 (Figure 3 and Table S1).

### Antimicrobial resistance profile of CR-hvKp isolates

**Figure 4.**
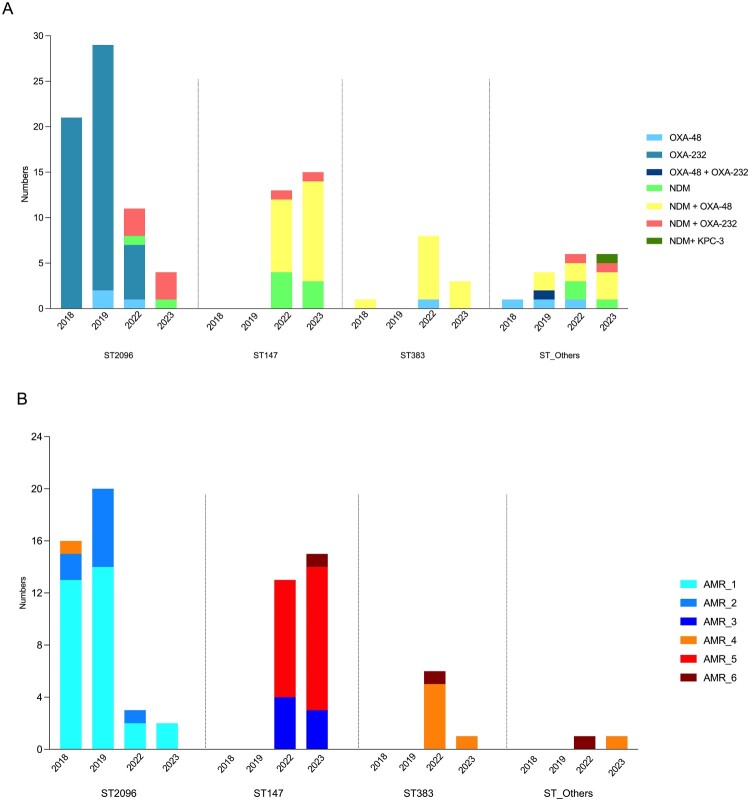
Temporal trends in antimicrobial resistance gene profiles among major CR-hvKp sequence types (2018–2023). (A) Distribution of carbapenemase genes among CR-hvKp isolates, stratified by sequence type. Bars represent the number of isolates carrying only *bla*_OXA-48_, only *bla*_OXA-232_, only *bla*_NDM_*, bla*_KPC-3_, or both genes. Colours indicate distinct carbapenemase profiles as shown in the legend (B) Distribution of chromosomal and plasmid-mediated antimicrobial resistance (AMR) gene profiles across major sequence types and years. Isolates were grouped into six AMR categories based on the combination of resistance genes detected: AMR_1: *aac(6’)_AGly, aadA_AGly, bla*_CTX_*, bla_OXA_, dfrA_Tmt, gyrA_Quin, ompK36_Carb, tet_TetCyc.* AMR_2: *AMR_1* + *pmrB_Col.* AMR_3: *aac(6’)_AGly, aadA_AGly, aph(3’)_AGly, bla_CTX_, bla_NDM_, dfrA_Tmt, gyrA_Quin, ompK36_Carb, pmrB_Col, qnrB_Quin.* AMR_4: *AMR_3* + *bla_OXA_, tet_TetCyc.* AMR_5: *AMR_4* – *tet_TetCyc.* AMR_6: *AMR_5* + *tet_TetCyc.* Colours indicate AMR categories as shown in the legend.

Analysis of antimicrobial resistance genes among CR-hvKp isolates revealed widespread carriage of multiple resistance determinants across sequence types (STs) (Table 1. and Figure 4). Among carbapenemase genes, *bla*_OXA-48_-like was the most prevalent, detected in 89.1% (114/128) of isolates, including *bla*_OXA-232_ in 53.9% (69/128) and *bla*_OXA-48_ in 35.9% (46/128). The *bla*_NDM_ gene was identified in 47.6% (61/128) of isolates. Co-carriage of *bla*_OXA-48_-like and *bla*_NDM_ was observed in 37.5% (48/128) of isolates (Figure 4A).

**Table 1.** Distribution of plasmid replicon types and AMR genes among CR-hvKp sequence types.

Replicon	Feature	ST2096 (*n* = 70),%	ST147 (*n* = 29),%	ST383 (*n* = 12),%	ST307 (*n* = 6),%	ST*_rare_* (*n* = 11),%	Total (*n* = 128), %
Plasmid replicons	Col	70 (100%)	5 (17%)	10 (83%)	5 (83%)	10 (90%)	100 (78%)
	IncA	1 (1%)	0 (0%)	0 (0%)	0 (0%)	0 (0%)	1 (0%)
	IncFIA	1 (1%)	0 (0%)	0 (0%)	0 (0%)	1 (9%)	2 (1%)
	IncFIB	3 (4%)	29 (100%)	0 (0%)	1 (16%)	7 (63%)	40 (31%)
	IncFIBK	67 (95%)	1 (3%)	1 (8%)	3 (50%)	5 (45%)	77 (60%)
	IncFIB_pNDM-MAR	70 (100%)	29 (100%)	12 (100%)	6 (100%)	11 (100%)	128 (100%)
	IncFII	2 (2%)	0 (0%)	1 (8%)	0 (0%)	3 (27%)	6 (4%)
	IncHI1B_pNDM-MAR	69 (98%)	29 (100%)	12 (100%)	6 (100%)	9 (81%)	125 (97%)
	IncI1	0 (0%)	0 (0%)	0 (0%)	0 (0%)	3 (27%)	3 (2%)
	IncL/M	7 (10%)	20 (69%)	12 (100%)	2 (33%)	8 (72%)	49 (38%)
	IncR	1 (1%)	0 (0%)	0 (0%)	0 (0%)	6 (54%)	7 (5%)
	IncX	1 (1%)	1 (3%)	1 (8%)	1 (16%)	0 (0%)	4 (3%)
AMR related genes	*aac(3)_AGly*	1 (1%)	0 (0%)	0 (0%)	1 (16%)	5 (45%)	7 (5%)
	*aac(6′)_AGly*	70 (100%)	29 (100%)	10 (83%)	6 (100%)	11 (100%)	126 (98%)
	*aadA_AGly*	69 (98%)	29 (100%)	10 (83%)	6 (100%)	9 (81%)	123 (96%)
	*ant(3″)_AGly*	0 (0%)	1 (3%)	0 (0%)	0 (0%)	0 (0%)	1 (0%)
	*aph(3′)_AGly*	10 (14%)	29 (100%)	12 (100%)	6 (100%)	7 (63%)	64 (50%)
	*dfrA_Tmt*	70 (100%)	29 (100%)	8 (66%)	6 (100%)	11 (100%)	124 (96%)
	*tet_TetCyc*	46 (65%)	1 (3%)	8 (66%)	4 (66%)	8 (72%)	67 (52%)
Mutations in resistance-related genes	*gyrA_Quin*	68 (97%)	29 (100%)	12 (100%)	6 (100%)	8 (72%)	123 (96%)
	*qnrB_Quin*	10 (14%)	29 (100%)	12 (100%)	6 (100%)	7 (63%)	64 (50%)
	*ompK36 GD*	68 (97%)	29 (100%)	0 (0%)	4 (67%)	7 (64%)	108 (84%)
	*ompK36* partial deletion	2 (3%)	0 (0%)	0 (0%)	1 (17%)	3 (27%)	6 (5%)
	*ompK35* partial p-deletion	11 (16%)	29 (100%)	12 (100%)	5 (83%)	11 (100%)	68 (53%)
	*phoQ_Col*	1 (1%)	0 (0%)	0 (0%)	0 (0%)	0 (0%)	1 (0%)
	*pmrA_Col*	0 (0%)	0 (0%)	0 (0%)	0 (0%)	0 (0%)	0 (0%)
	*pmrB_Col*	14 (20%)	29 (100%)	1 (8%)	0 (0%)	4 (36%)	48 (37%)
Carbapenemases and β-lactamases	*bla_CTX_*	63 (90%)	29 (100%)	12 (100%)	6 (100%)	11 (100%)	121 (94%)
	*bla_GES_*	0 (0%)	0 (0%)	0 (0%)	0 (0%)	0 (0%)	0 (0%)
	*bla_KPC_*	0 (0%)	0 (0%)	0 (0%)	1 (16%)	0 (0%)	1 (0%)
	*bla_NDMs_*	8 (12%)	29 (100%)	11 (91%)	5 (83%)	10 (91%)	61 (48%)
	*bla_OXA-48_-like*	67 (95%)	22 (75%)	12 (100%)	2 (33%)	11 (100%)	114 (89%)

Values indicate number of isolates (percentage).

*ST*_rare_* refers to sequence types represented by only one or two isolates within the dataset.

In ST147 isolates, *bla*_NDM_ (identified as *bla*_NDM-5_) was detected in all isolates (100%). Within this group, *bla*_OXA-48_ was the predominant variant, detected in 68.9% (20/29) of isolates, while *bla*_OXA-232_ was identified in two isolates. Co-occurrence of *bla*_NDM_ with *bla*_OXA-48_ was observed in 75.9% (22/29) of ST147 isolates. In contrast, among ST2096 isolates, *bla*_OXA-48_-like were detected in 95.7% (67/70), dominantly *bla*_OXA-232_ (91.4%, 64/70), whereas *bla*_OXA-48_ was detected in 4.2% (3/70). The *bla*_NDM_ was present in 11.4% (8/70) of ST2096 isolates, with co-carriage of *bla*_NDM_ and *bla*_OXA-48_ observed in 8.5% (6/70). One ST307 isolate co-carried *bla*_NDM-5_ *bla*_KPC-3_. Among CR-hvKp isolates collected between 2018 and 2019, exclusively *bla*_OXA-48_-like and predominantly *bla*_OXA-232_ were carried, whereas co-positivity for *bla*_NDM_ with *bla*_OXA-48_ or *bla*_OXA-232_ first emerged in both ST147 and ST2096 after 2022. In ST2096 isolates, all co-positive cases involved *bla*_NDM_ together with *bla*_OXA-232_. In ST147 isolates, co-positive included both *bla*_OXA-48_ and *bla*_OXA-232_, with most cases involving the *bla*_NDM_ – *bla*_OXA-48_ combination. (Figure 4A; Table S1). Extended-spectrum beta-lactamase gene *bla*_CTX-M_ was detected in 94% (121/128) of isolates, with 100% prevalence in ST147, ST383, ST307, and ST*_rare_* isolates (Table 1).

Regarding aminoglycoside resistance, the aminoglycoside-3′-phosphotransferase gene (*aph(3*′*))* gene was present in 50% (64/128) of isolates. It was detected in all ST147, ST383, and ST307 isolates, but only in 14.3% (10/70) of ST2096. The aminoglycoside 6′-N-acetyltransferase gene (aac(6′)) and aminoglycoside adenylyltransferase gene (*aadA*) were nearly ubiquitous across all STs (98–100%). In ST2096, the trimethoprim resistance *dfrA* gene was present in all isolates (100%, 70/70). The quinolone resistance *qnrB* gene was found in 50% (64/128) of isolates, including all ST147 (100%, 29/29) and a small subset of ST2096 (14.3%, 10/70). Tetracycline resistance *tet* resistance genes were present in 52.3% (67/128), with high prevalence in ST2096 (65.7%, 46/70) and ST rare (72.7%, 8/11), but were uncommon in ST147 (3.4%, 1/29) (Table 1 and Supplementary Table S1).

Colistin resistance-related *pmrB* mutations were found in 37.5% (48/128) of isolates, present in all ST147 isolates and in 20% (14/70) of ST2096. No mutations were identified in *pmrA* (Table 1). Partial deletions in *ompK35* were detected in 68 of 128 (53.1%) CR-hvKp isolates, whereas the *ompK36* GD mutation was present in 84% (108/128) isolates. Both alterations co-occurred in 40.6% (52/128) isolates. Notably, all ST147 isolates harboured both *ompK35* partial deletion and the *ompK36* GD mutation, while this combination was observed in 14.2% (10/70) ST2096 isolates. Mutations in *gyrA* associated with fluoroquinolone resistance were detected in 96% (123/128) isolates (Table 1 and Table S1).

Analysis of AMR gene profiles over time revealed distinct resistance evolution patterns across major CR-hvKp sequence types (Figure 4B). The majority of ST2096 isolates belonged to the AMR_1 category, followed by a smaller proportion in AMR_2. In 2023, all ST2096 isolates were classified within the AMR_1 group. In contrast, ST147 showed a broader resistance spectrum, with most isolates falling into AMR_5 and AMR_3 categories. In 2023, two ST147 isolates carried the most extensive resistance profile, classified as AMR_6. For ST383, most isolates were grouped within the AMR_4 category (Figure 4B).

### Plasmid profiles of CR-hvKp and the hybrid plasmid structure of ST147

Plasmid replicon typing revealed that all CR-hvKp isolates harboured the IncFIB_pNDM-MAR replicon regardless of sequence type (ST) (Table 1). Similarly, IncHI1B_pNDM-MAR was detected in 97.7% (125/128) of isolates, including all of ST147, ST383, ST307, and 98.6% of ST2096 (69/70). The Col replicon was highly prevalent in ST2096 (100%, 71/71), ST307 (83.3%, 5/6), ST383 (83.3%, 10/12), but rare in ST147 (17.2%, 5/29). In contrast, IncFIB was present in all ST147 isolates (100%, 29/29) but was infrequent in ST2096 (4.3%, 3/70) and absent in ST383. IncFIBK was commonly detected in ST2096 (94.3%, 67/71) and moderately present in ST307 (50%, 3/6), but rare in ST147 (3.4%, 1/29). The IncL/M replicon was found in all ST383 isolates (100%, 12/12), and ST147 (69%, 20/29), but in 9.8% of ST2096 (7/71). Other replicons, such as IncA, IncFIA, IncFII, and IncR, were rarely detected (Table 1.). No isolate carried IncN or IncQ replicons (Table S1).

**Figure 5.**
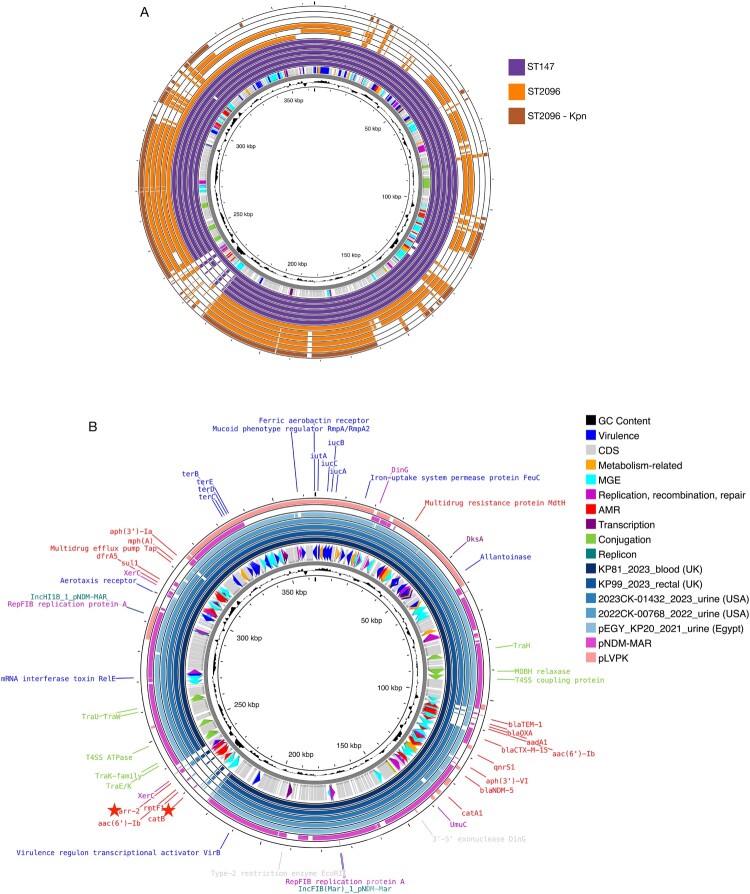
A. Comparison of PF302 hybrid plasmid to other multireplicon plasmids from ST147 and ST2096 isolates. ST2096 – Kpn represents an isolate with three replicons: IncFIB(K), IncFIB(Mar), and IncHI1B. B. The comparison of PF302 hybrid plasmid to the closest matches in the PLSDB, as well as the pNDM-MAR and pLVPK backbones. Main features on the hybrid plasmid are colour-coded as given in the legend.  Colours indicate functional gene categories and plasmid features as defined in the legend for the pPF302 hybrid plasmid from the representative ST147 isolate. The pPF302 hybrid plasmid-specific region includes an in-depth analysis of mobility-related elements, given as a separate track indicated by stars.

In long-read sequencing, within ST147 (6/6) isolates, IncFIB(Mar) and IncHI1B replicons from pNDM-MAR (Genbank ID: JN420336) were found together in a single contig of ∼348-364 Mb in length. In ST2096, 3 isolates also carried IncFIB(Mar) and IncHI1B replicons on a single plasmid, considerably smaller than the ST147 isolates (∼202-296 Mb) (Figure 5A). Two ST2096 isolates had these replicons on separate contigs. Another ST2096 isolate carried a plasmid that contained a third replicon IncFIB(K), in addition to IncFIB(Mar) and IncHI1B; this plasmid was also relatively smaller (∼294 Mb) (Figure 5A). Despite carrying the same replicons, multireplicon plasmids carried by ST147 and ST2096 differed. The multireplicon plasmids from three ST147 isolates were almost identical to each other (PF149, PF573 and PF768) despite being distant based on whole genome analysis (Table S3).

Compared with ST147, the multireplicon plasmids identified in ST2096 isolates carried fewer antimicrobial resistance genes, and aerobactin-associated genes were absent from the plasmids in most ST2096 isolates (4/6) (Figure 5A and Table S3). Additionally, despite the presence of the replicons from pNDM-MAR, ST2096 multireplicon plasmids lacked the *bla_NDM_* gene. In contrast, all but one ST147 multireplicon plasmid had the *bla_NDM-5_* variant, suggesting a hybrid plasmid structure. These hybrid plasmids were predicted to be conjugative, carrying a MOBH relaxase (relaxase or Mob for mobilisation protein) and an F-type conjugation system. The MOBH relaxase was apparently lost from 3 of the ST2096 multireplicon plasmids, two of which also lacked the conjugation system genes (Table S3). Multireplicon plasmids from all ST147 isolates carried tellurium resistance genes, whereas those from ST2096 carried mercury resistance genes (4/6), including one plasmid that also harboured tellurium resistance genes, similar to pNDM-MAR. In addition to the multireplicon/hybrid plasmids, ST147 isolates carried plasmids with IncFIB (pKPHS1 or pQil) or IncL/M(pOXA-48) replicons (Figure S3), while ST2096 isolates carried plasmids with ColKP3 or lncFIB(K) plasmids. ST147 isolates exclusively carried *bla*_OXA-48_ on IncL/M plasmids (Figure S3B). ST2096 isolates, however, typically carried *bla*_OXA-1_ and *bla*_OXA-232_ variants mostly on ColKP3 plasmids.

The multireplicon plasmids from ST147 isolates, carrying IncFIB(Mar) and IncHI1B, had a hybrid plasmid structure combining both resistance and virulence genes (Figure 5B and Figure S3). The hybrid plasmids shared the majority of the backbone from pNDM-MAR especially around the antimicrobial resistance genes, together with the virulence gene block that was also present in the known virulence plasmid pLVPK (NC_005249.1), including aerobactin genes and the mucoid phenotype regulator (Figure 5B). Resistance determinants typically included multiple β-lactamases (TEM-1, OXA-9, CTX-M-15, NDM-5) and aminoglycoside-modifying enzymes (aadA1, aac(6′)-Ib, aph(3′)-VI, aac(6′)-Ib, aph(3′)-Ia), chloramphenicol acetyltransferases, dihydropteroate synthase sul1, dihydrofolate reductase type 1, bleomycin resistance protein, quinolone resistance pentapeptide repeat protein qnrS1, and multiple multidrug efflux systems (MdtH, EmrE, Tap) (Figure 5B and Table S3). One hybrid plasmid from an ST147 isolate, pPF302_hybrid, had a small segment with additional resistance genes, chloramphenicol resistance gene *catB*, an ADP-ribosyltransferase *arr-2 (*rifampicin resistance related gene*)* and a 16S rRNA methyltransferase *rmtF1* (Figure 5B). PF302 isolate was also phenotypically resistant to rifampicin. A search of the plasmid database PLSDB identified close matches to pPF302_hybrid. The top matches were from the UK, USA, and Egypt, isolated between 2021 and 2023, and all lacked this small segment (Figure 5B). This region was also absent in pNDM-MAR or PLVPK. The virulence gene block was mostly shared between pPF302_hybrid and closest matches from PLSDB. The mucoid phenotype regulator gene *rmpA/rmpA2* was detected in pPF302_hybrid, as well as hybrid plasmids from our other ST147 isolates, but not in pNDM-MAR or the Egypt ST147 isolate from 2021 (pEGY_KP20_2021) (Figure 5B).

## Discussion

The integration of resistance and virulence traits in CR-hvKp underscores the urgent need to elucidate the reasons underlying their dissemination. In this study, we performed a comprehensive genomic analysis of CR-hvKp isolates collected across Türkiye where the burden of antibiotic resistance is high. Our findings revealed an increase in CR-hvKp incidence, accompanied by a shift towards high-risk clones over the years. ST147 isolates harboured a hybrid plasmid carrying IncFIB and IncHI1B replicons related to the pNDM-MAR plasmid. This plasmid also contained a virulence gene island typically associated with virulence plasmids, such as pLVPK, thereby combining resistance and virulence determinants within a single conjugative structure. Specifically, one ST147 isolate (pPF302_hybrid) carried additional resistance determinants on this hybrid plasmid, *rmtF1* and *arr-2*, which were absent in the pNDM-MAR backbone.

The global prevalence of CR-hvKp has risen substantially, with reported rates ranging from 7.8% to over 40% in clinical isolates and exceeding 50% in some regions particularly China and Southeast Asia [1,4,6]. In our study, the proportion of hvKp among CR-Kp isolates increased from 20.7% in 2018 to 33.1% by 2023. The definition of “hypervirulence ” is still challenging, particularly in epidemiological comparisons and clinical interpretations [34]. Given this heterogeneity, we applied a genomically defined hvKp framework rather than a phenotypic definition. Aerobactin (*iucA*) and regulators of the mucoid phenotype (*rmpA/rmpA2*) have been shown to exhibit high diagnostic accuracy in distinguishing hvKp-enriched populations from classical *K. pneumoniae*, outperforming available phenotypic assays in large strain collections [35]. In addition, experimental deletion of these loci (Δ*rmpA*Δ*iucA*) resulted in significantly increased LD₅₀ values in animal models [19]. Based on this, we defined hvKp with the co-existence of *rmpA/rmpA2* and *iucA/iutA* loci as core virulence markers to minimise ambiguity and ensure consistency in large-scale genomic surveillance studies [14].

We found that ST2096 was the dominant CR-hvKp clone between 2018 and 2019, accounting for over 90% of isolates during that period. In this clone, *bla*_OXA-48_-like were detected in 95.7% (67/70) dominantly *bla*_OXA-232_ (91.4%, 64/70) of ST2096 isolates while *bla*_NDM_ was present in 11.4% (8/70). We previously reported the same carbapenemase profile in a national surveillance study from Türkiye [15]. Following 2022, a marked clonal shift was observed, with the majority of the CR-hvKp isolates belonged to ST147, harbouring *bla*_NDM-5_, either alone or together with *bla*_OXA-48_-like*.* The *bla*_NDM-5_ has been shown to enhance bacterial fitness under zinc starvation and host immune pressure [30]. ST147, initially characterised as a carbapenem-resistant nosocomial lineage, has recently evolved into a dual-risk clone via acquisition of virulence plasmids. It has been linked to outbreaks across Europe, Iran and Latin America and is increasingly recognised as a pandemic-threat lineage [15,36,37].

The two clones also differed in the plasmid backbones carrying carbapenemase genes. ST147 isolates exclusively carried *bla*_OXA-48_ on IncL/M plasmids, whereas ST2096 isolates typically carried *bla*_OXA-232_ variants mostly on ColKP3 plasmids. The *bla*_OXA-48_ gene is most commonly associated with the composite transposon Tn1999.2 located on IncL/M-type conjugative plasmids, which have been widely reported in Europe and Asia [38]. Notably, Tn1999.2 was first described in Türkiye between 2006 and 2007 and was characterized by the presence of an IS1R insertion upstream of *bla*_OXA-48_, generating a strong hybrid promoter and increased carbapenemase expression [16]. In contrast, *bla*_OXA-232_ has been predominantly linked to small, mobilisable ColKP3-type plasmids and has been frequently reported from India and surrounding regions [38], consistent with the dissemination pattern observed among ST2096 isolates in our cohort. Our findings highlight a temporal and lineage-specific shift in CR-hvKp in Türkiye. The co-carriage of *bla*_NDM_ and *bla*_OXA-48_-like carbapenemases suggests the cross-border transmission of resistance and virulence.

The distribution of CR-hvKp clones also shows regional variation. ST23-K1 is common in community-acquired cases in China, while ST15 and ST65 have also been detected in several Asian countries [2,9]. In line with previous reports, we also observed regional CR-HvKp distribution in our cohort, with ST2096 predominating in İstanbul (43.6%) and ST147 being the most common in the Aegean region (55.2%). The geographic variation in ST distribution observed in this study suggests region-specific clonal dynamics. Stratification by resistance profiles revealed that ST147 isolates carried a higher resistance score (AMR_6) than ST2096 (AMR_2). This finding suggests that ST147 may have enhanced adaptive potential facilitating its persistence and dissemination under sustained antimicrobial pressure, while regional differences in antimicrobial usage, particularly carbapenem exposure, and variability in infection control practices across hospitals may further shape the persistence or replacement of predominant clones in different regions (Figure 4).

The genomic analysis showed that 79.7% (102/128) of CR-hvKp isolates harboured the chromosomal yersiniabactin *(ybt)* locus, whereas colibactin (*clb*) was detected in only three isolates and salmochelin *(iro)* was absent (Figure 2, Table S1). Similarly, a recent study from China reported CR-hvKp carrying pLVPK-like plasmids with deletions in the *iroBCDN* cluster [39]. The absence of *iro* and the low frequency of *clb* among our isolates may therefore reflect the circulation of virulence plasmids with partial or truncated gene content in Türkiye. In addition, all ST147 and 97.1% (68/70) of ST2096 CR-hvKp isolates carried the KL64 capsular serotype, which has previously been linked to high virulence and mortality in ST11 [40]. The enhanced antioxidant capacity conferred by KL64 is thought to improve bacterial persistence within macrophages [41]. The widespread presence of this capsule among our predominant clones is suggestive of its role in the positive selection of hypervirulent lineages.

Large-scale analyses have identified two dominant IncFIB lineages, IncFIB(K) and IncFIB(Mar), as the principal backbones driving CR-hvKp dissemination across Asia and Europe [42]. In our collection, IncFIB was detected in nearly all ST147 isolates, while IncFIB(K) was found in only 3%. In contrast, ST2096 isolates displayed the opposite trend, with IncFIB(K) highly prevalent (95.7%) and IncFIB detected in Table 1 . This pattern suggested clone-specific selectivity of replicons in CR-HvKp. The ST147 isolates harbouring IncHI1B and two distinct IncFIB replicons linked with a wide array of resistance genes were also reported [43,44].

Long-read sequencing of ST147 and ST2096 isolates revealed that both sequence types harboured plasmids containing IncFIB(Mar) and IncHI1B replicons related to pNDM-MAR within a single plasmid structure. In ST2096, these multireplicon plasmids lacked *bla*_NDM_ genes, whereas in ST147, the majority of multireplicon plasmids (5/6) carried *bla*_NDM-5_ on an IncFIB(pQil) backbone. Recent studies have indicated that the locali zation of *bla*_NDM_ in ST147 is variable, with some isolates harbouring it on hybrid plasmids, whereas others retain it on distinct plasmids[44]. Additionally, ST2096 multireplicon plasmids lacked the virulence gene island (Figure 5). In contrast, ST147 multireplicon plasmids represented a true hybrid structure, bringing together resistance determinants from the pNDM-MAR backbone and virulence determinants likely from pLVPK or a related backbone. Turton et al. were first identified as a hybrid resistance/virulence plasmid in an ST147 (KL20) strain, which also carried *bla*_NDM-1_ on a separate plasmid [44]. Turton et al. also reported that ST147 isolates with a capsular type of KL64 carry *bla*_NDM-5_ on hybrid plasmids [44]. In our study, the ST147 isolates carrying *bla*_NDM-5_ on IncFIB(pQil) had the KL64 capsule type.

We also identified one ST147 isolate with additional resistance genes on the hybrid plasmid (pPF302_hybrid), *rmtF1* (a 16S rRNA methyltransferase) and *arr-2*. These genes have not been previously reported in other ST147-associated hybrid plasmids [14,44] and were not detected in the closest matching ST147 hybrid plasmids available in the PLSDB database. The *rmtF1* mediates high-level resistance to aminoglycosides, while *arr-2*, typically embedded within class 1 integrons, confers resistance to rifampicin [45,46]. These resistance determinants were also detected on IncFIB(pQil)-type plasmids in ST147 isolates. The acquisition of such resistance genes by hybrid plasmids in carbapenem-resistant ST147 strains suggests an expansion of their antimicrobial resistance repertoire, which may further compromise the effectiveness of combination therapy strategies commonly used in clinical practice.

The limitation of the study is that the classification of HvKp was done based on genomic virulence markers. The lack of phenotypic assays, in vivo validation, and clinical outcome correlation may not reflect the hypervirulent behaviour of the strains.

## Conclusion

Our genomic analysis highlights the expanding threat posed by CR-hvKp in Türkiye. We observed a shift from ST2096 to ST147 as the major CR-hvKp lineage after 2022. This transition might have been facilitated by the hybrid plasmids pNDM-MAR and IncL/M plasmids together with virulence genes. Of particular concern, we identified an ST147 isolate with additional resistance determinants (*rmtF1* and *arr-2*) on the hybrid plasmid, not present in the best matching ST147 hybrid plasmids from the PLSDB. Our results not only confirm ST147 as an emerging pandemic-threat lineage in Türkiye but also highlight the urgent need for genomic surveillance and novel diagnostic strategies to prevent the dissemination of CR-hvKp.

## Supplementary Material

SupplementaryTable_S1.xlsx

Supplementary Figure S2_map.png

SupplementaryTable_S2.xlsx

Supplementary Figure S1_revised_hvkp_m1.jpeg

SupplementaryTableS3.xlsx

Supplementary Figure_3.pdf

## Data Availability

Whole-genome sequencing data from the THREAT study (NCT03597841) are available in the NCBI BioProject database under accession number PRJNA789336. Plasmid sequence data generated in this study have been made publicly available via Figshare (https://figshare.com/s/12af884490e6c45c1ee9).
