# Supplementary figures and images for: Emergence of carbapenem-resistant putative hypervirulent Klebsiella pneumoniae ST147 with a distinct hybrid plasmid in Türkiye
Source: Emerg Microbes Infect. 2026 Mar 31;15(1):2653280. doi: 10.1080/22221751.2026.2653280 (PMC13084844; doi:10.1080/22221751.2026.2653280)

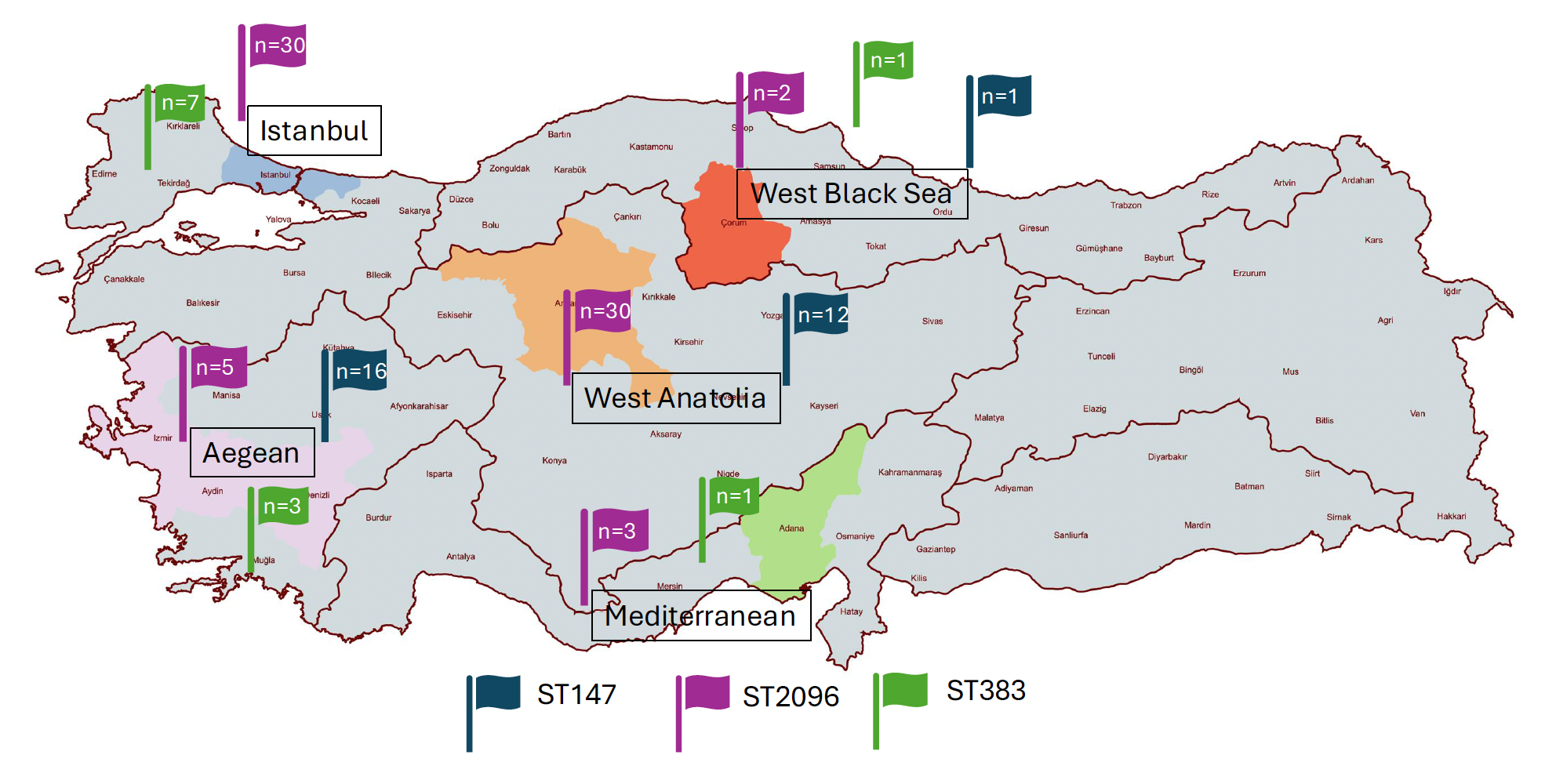

Supplement: Supplementary Figure S2_map.png [file TEMI_A_2653280_SM8333.png]

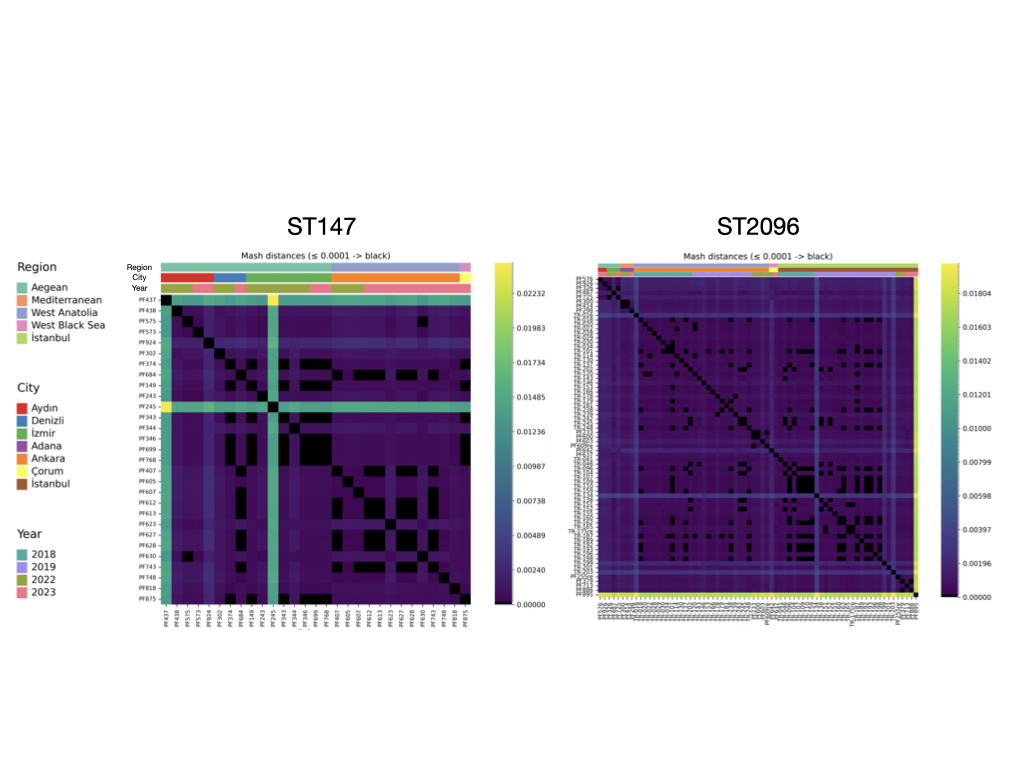

Supplement: Supplementary Figure S1_revised_hvkp_m1.jpeg [file TEMI_A_2653280_SM8331.jpeg]

A

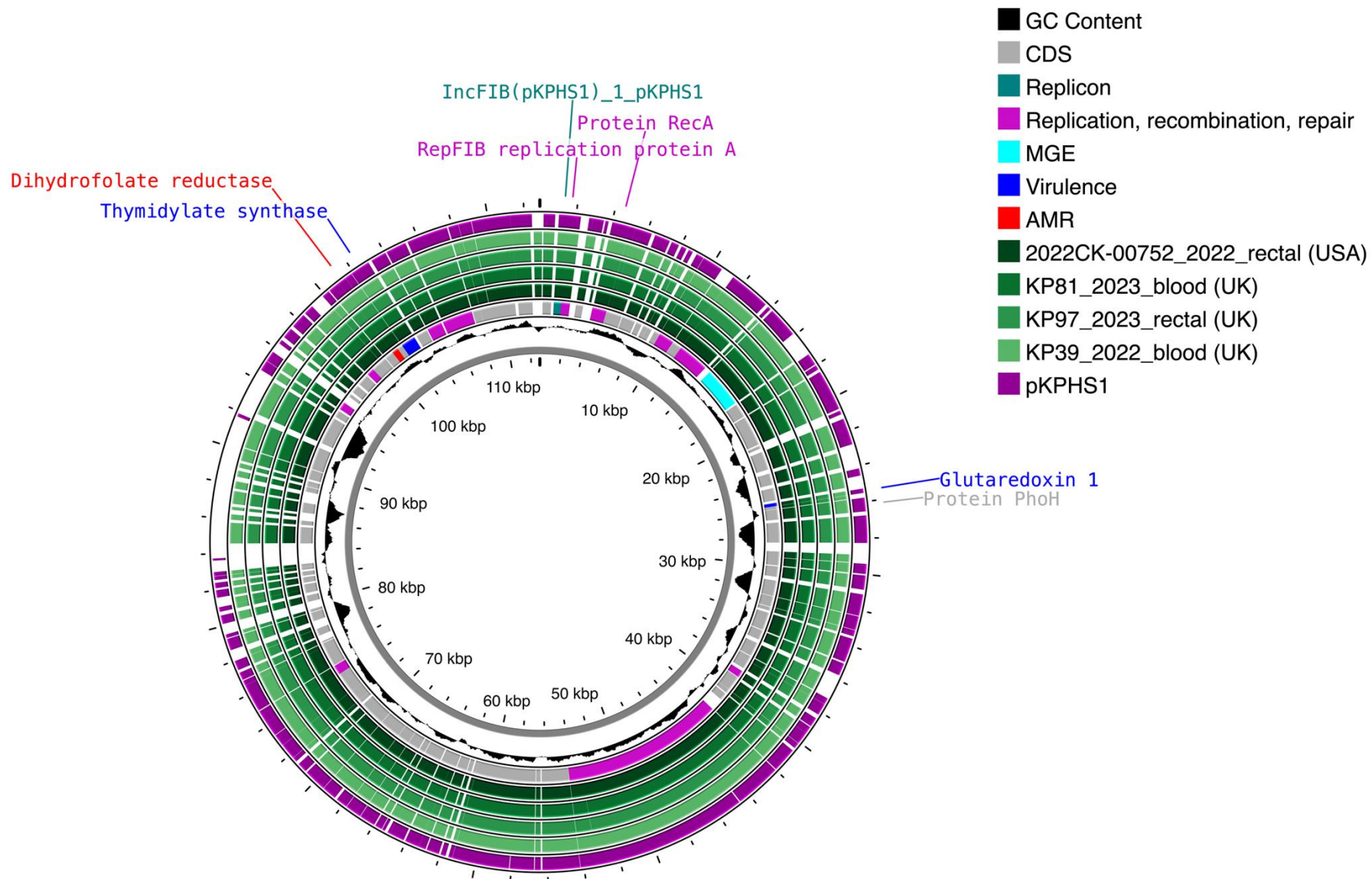

B

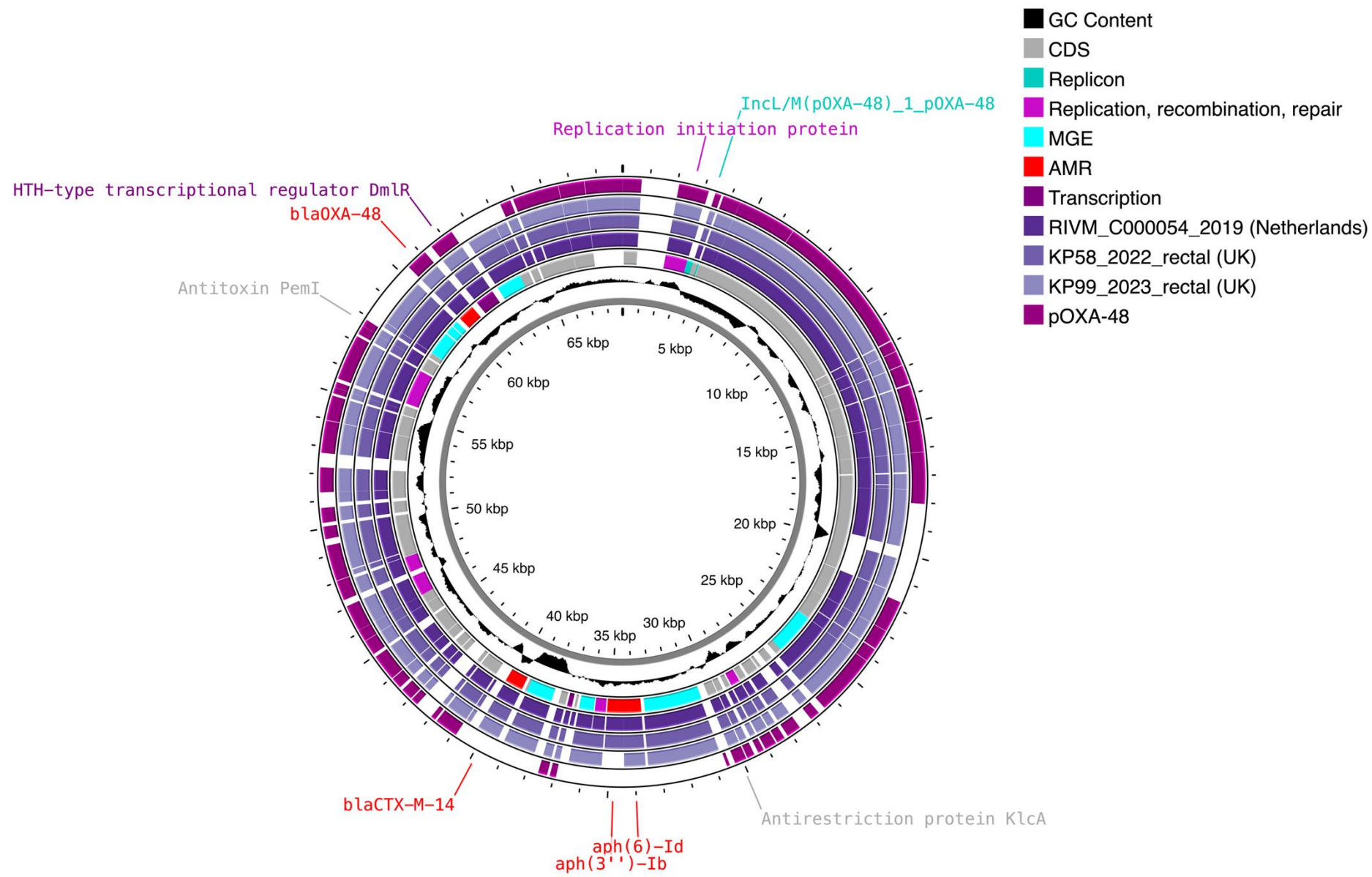

Supplement: Supplementary Figure_3.pdf [file TEMI_A_2653280_SM8329.pdf]
